# Ionic Conductivity of Electrolytes Composed of Oleate-Capped Yttria-Stabilized Zirconia Nanoparticles

**DOI:** 10.1021/acsomega.3c05368

**Published:** 2023-12-07

**Authors:** Yuki Makinose, Tetsuya Yamada, Yuta Kubota

**Affiliations:** † Graduate School of Natural Science and Technology, Shimane University, 1060 Nishikawatsu-cho, Matsue 690-8504, Japan; ‡ Laboratory for Future Interdisciplinary Research of Science and Technology, Tokyo Institute of Technology, 4259 Nagatsuta, Midori, Yokohama, Kanagawa 226-8503, Japan; § Department of Materials Science and Engineering, School of Materials and Chemical Technology, Tokyo Institute of Technology, 2-12-1 Ookayama, Meguro, Tokyo 152-8550, Japan

## Abstract

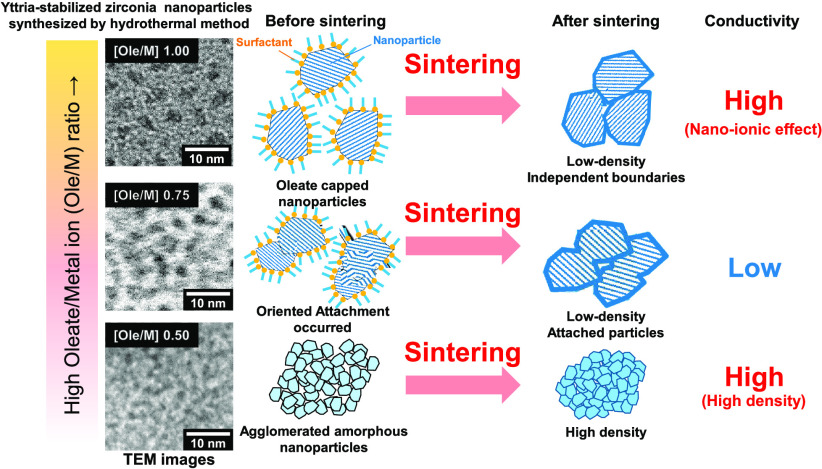

Yttria-stabilized zirconia (YSZ) is a highly promising electrolyte material for solid oxide fuel cells (SOFCs). We investigated the conductivity-enhancing effect of nanosized YSZ to explore key techniques to decrease the operating temperature. YSZ nanoparticles ranging from 2 to 4 nm were synthesized with oleate groups by the hydrothermal method at various oleate/metal ion ratios (Ole/M = 1.00, 0.75, and 0.50). The nanoparticles were sintered, and the ionic conductivities were evaluated. The 1.00 Ole/M sample exhibited high dispersibility in cyclohexane and showed a nearly monodispersed distribution. The other samples possessed agglomerated nanoparticles. The sintered YSZ nanoparticles had densities of 3.36–2.80 g/cm^3^ and ionic conductivities of 2.52–1.16 mS/cm at 750 °C, which are higher than those of commercial 8 mol % YSZ. Furthermore, the sintered YSZ nanoparticles exhibited higher activation energies than the commercial samples in the lower temperature range (550–650 °C). The ionic conductivity enhancement despite the high activation energy is likely due to the increased grain boundary volume. This study demonstrated the successful production of YSZ with high ionic conductivity and sinterability upon sintering at 1050 °C using YSZ nanoparticles.

## Introduction

Yttria-stabilized zirconia (YSZ) is a highly promising electrolyte material for solid oxide fuel cells (SOFCs) because of its exceptional oxide ionic conductivity, chemical stability, and mechanical resistance. SOFCs are flexible electrochemical energy converters capable of utilizing various types of fuels, including hydrogen, ammonia, ethanol, and lignin.^1^ Additionally, SOFCs have low greenhouse gas emissions owing to their high efficiency (surpassing 70%) and a long operational lifetime of over 4000 h.^2^

However, the widespread implementation of SOFCs has been hindered by the high production cost of electrolyte, electrode, and catalyst materials. In particular, the high operating temperature of SOFCs (800–1000 °C) induces challenges such as interfacial diffusion issues between a solid electrolyte and electrodes, catalyst poisoning, thermal heat shock, and differences in thermal expansion coefficients among cell components.^3^ These limitations restrict the choice of materials suitable for high-temperature SOFCs. Additionally, the high operating temperature of SOFCs is attributed to the electrolytes. YSZ is commonly used as a solid electrolyte in high-temperature SOFCs because of its high oxide ionic conductivity. However, YSZ exhibits low ionic conductivity at low-to-intermediate operating temperatures (400–800 °C).^4^

To reduce the operating temperature, one possible approach is to reduce the particle size to the nanoscale and utilize the nanoionic effect, enhancing ion transport using nanoscale materials. Various studies have been conducted on nanosized YSZ with the aim of reducing the operating temperature of the SOFC. For instance, in the previous work, YSZ with a crystal grain size of around 20 nm exhibited a higher conductivity than polycrystalline YSZ with microsize grains.^5^ Furthermore, nanoparticles of size 20 to 30 nm using the sol–gel method showed a conductivity of 0.107 S/cm at 1200 °C, suggesting an effect due to the grain boundaries at the nanoscale.^6^

On the other hand, there is a controversy over the nanoionic effect. For example, the relationship between grain size and conductivity was explored using milled YSZ nanoparticles; YSZ nanoparticles with grains around 17 nm showed a lower conductivity than those with grains of approximately 50 nm.^7^ In addition, the synthetic process of materials and the difference of evaluation could lead to different results. The prevailing proposals for explaining the nanoionic effect are strain in the nanoscale, degree of crystallinity, space charge, and grain size. However, a common explanation of the nanoionic effect has not been achieved.^8^

Herein, we investigated the effect of the particle size of YSZ before sintering on ionic conductivity. First, YSZ nanoparticles (NPs) ranging from 2 to 4 nm were synthesized using the hydrothermal method by using sodium oleate as a surfactant. The particle size obtained in this study is smaller than that obtained in a previous study^5,7^ and it exhibited high dispersibility because of the surfactant. Subsequently, the YSZ nanoparticle samples with different sizes were sintered at 1050 °C. The sintered YSZ pellets were characterized by X-ray diffraction (XRD) and scanning electron microscopy (SEM). Finally, the ionic conductivity of the sintered pellet with initial different particles sizes was evaluated. The effect of particle size (presintering) on ionic conductivity was discussed in terms of grain size and relative density.

## Methods

### Source Materials

ZrOCl_2_·8H_2_O (Fujifilm Wako Pure Chemical Corp., Wako Special Grade), YCl_3_·6H_2_O (Fujifilm Wako Pure Chemical Corp., 99.9%), sodium oleate (Nacalai Tesque Inc., Extra Pure Reagent), 28% ammonia (NH_3_) aqueous solution (Fujifilm Wako Pure Chemical Corp., Guaranteed Reagent), and 3 and 8 mol % YSZ (Tosoh Cop., TZ-3Y-E and TZ-8YS, both 40 nm in size) were used as the source materials.

### Synthesis

YSZ nanoparticles (NPs) were prepared using an oleate-modified hydrothermal growth method.^9−11^ A metal ion solution was prepared by mixing ZrOCl_2_ (4.60 mmol) and YCl_3_ (0.80 mmol) salts with 10 mL of distilled water. Sodium oleate solution was prepared by mixing sodium oleate with 20 mL of distilled water. The amount of sodium oleate was varied at 5.00, 3.75, and 2.50 mmol, corresponding to the [oleate ion/(Y+Zr) ions] (Ole/M) ratios of 1.00, 0.75, and 0.50, respectively. Sodium oleate solution was added dropwise to the metal ion solution under stirring. Then, 5 mL of NH_3_ was added to the mixture. The mixture was transferred to a poly(tetrafluoroethylene) (PTFE) vessel and placed inside a stainless-steel autoclave. The hydrothermal method was conducted at 200 °C for 6 h in an electric oven. The obtained samples were centrifuged at 5500 rpm for 5 min, washed with water, and dried at 60 °C for 20 h.

The YSZ NPs were subjected to presintering via heat treatment at 600 °C for 20 min. The prepared YSZ powder was washed several times with distilled water and ethanol and dried overnight at 25 °C. A total of 250 mg of YSZ powder was formed into a pellet with a diameter of 14 mm at 200 MPa. The pellet was sintered at 1050 °C for 2 h. Commercial YSZ (3 and 8 mol % Y_2_O_3_, referred to as 3YSZ and 8YSZ, respectively) was also pressed and sintered; it was used as a reference sample. A total of 500 mg of powder was used to form commercial pellets.

### Characterization

YSZ NPs and sintered YSZ were characterized through powder XRD conducted on a Smart Lab XE system (Rigaku, Japan) using Cu Kα radiation. XRD was performed by using a silicon sample holder (low background). Transmission electron microscopy (TEM) was conducted by using a JEM-2010 microscope (JEOL, Japan). The sample dispersed in cyclohexane was dropped onto an elastic carbon-support film, and NP size distributions were determined from TEM images using ImageJ software.^12^ The selected area electron diffraction (SAED) patterns were analyzed using ReciPro software.^13^ Energy-dispersive X-ray (EDX) spectroscopy was carried out using an EDX-900 (Shimadzu, Japan) instrument. The density was measured using the apparent bulk density. It was calculated based on simple diameter, thickness, and weight, without considering the porosity.

For electrode preparation, Ag was sputter-deposited on both sides of the sintered samples. Subsequently, Ag paste (D–500, Fujikura Kasei, Japan) was applied on sputtered Ag so that it could be used as the collector. The conductivity of YSZ was measured through impedance spectroscopy (Wave Driver 100 EIS Potentiostat/Galvanostat, PINE Research) conducted in the range of 1 MHz to 1 Hz at temperatures of 550–900 °C in air.

## Results

**Figure 1:**
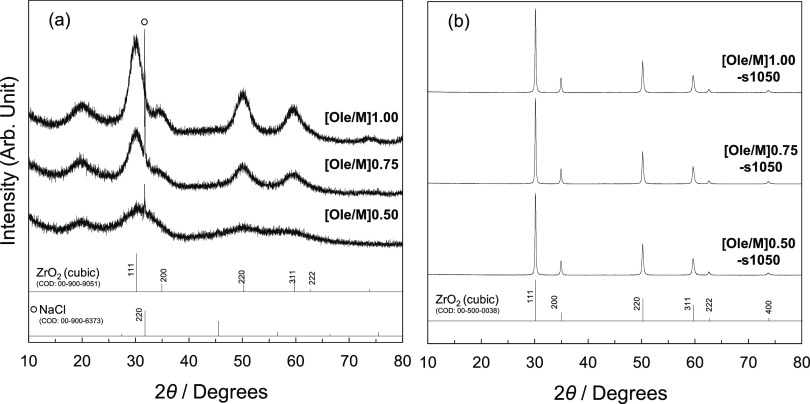
XRD patterns of (a) YSZ NPs synthesized via the hydrothermal method and (b) YSZ sintered at 1050 °C.

XRD patterns were analyzed to identify the crystal phases of the NPs. Figure 1 shows the XRD patterns of the YSZ NPs synthesized via the hydrothermal method and of sintered YSZ with various oleate/metal ion (Ole/M) ratios. The patterns of the NPs corresponded to cubic ZrO_2_ (COD 00-900-9051). The crystallite sizes of the samples synthesized via the hydrothermal method were calculated using the Debye–Scherrer equation and Gauss fitting from the 220 diffraction peak. The sizes of YSZ NPs with Ole/M ratios of 1.00, 0.75, and 0.50 were 6.3 nm, 5.3 nm, and incalculable, respectively. The crystallite size increased with the increasing Ole/M ratio, which is a trend opposite to that reported in a previous study.^4^ Oleate ions are considered “inhibitors” for crystal growth. In addition, the pH values were 10.42, 10.36, and 10.29 at Ole/M ratios of 1.00, 0.75, and 0.50, respectively, before the hydrothermal method. This is because sodium oleate increased the pH of the reaction solution. High pH affects the acceleration of crystal growth.

Furthermore, the NaCl phase was detected in the samples, which was difficult to eliminate because of the decrease in dispersibility caused by repeated water washing. The Y_2_O_3_ mol % compared to ZrO_2_ + Y_2_O_3_, as detected by EDX measurements, was 7.8, 8.0, and 7.7 mol % at the Ole/M ratios of 1.00, 0.75, and 0.50, respectively. The patterns of the sintered YSZ NPs also corresponded to those of cubic ZrO_2_. The NaCl phase was nearly eliminated from the sintered samples upon washing. The crystallite sizes of the sintered YSZ NPs with Ole/M ratios of 1.00, 0.75, and 0.50 were 56, 57, and 55 nm, respectively.

**Figure 2:**
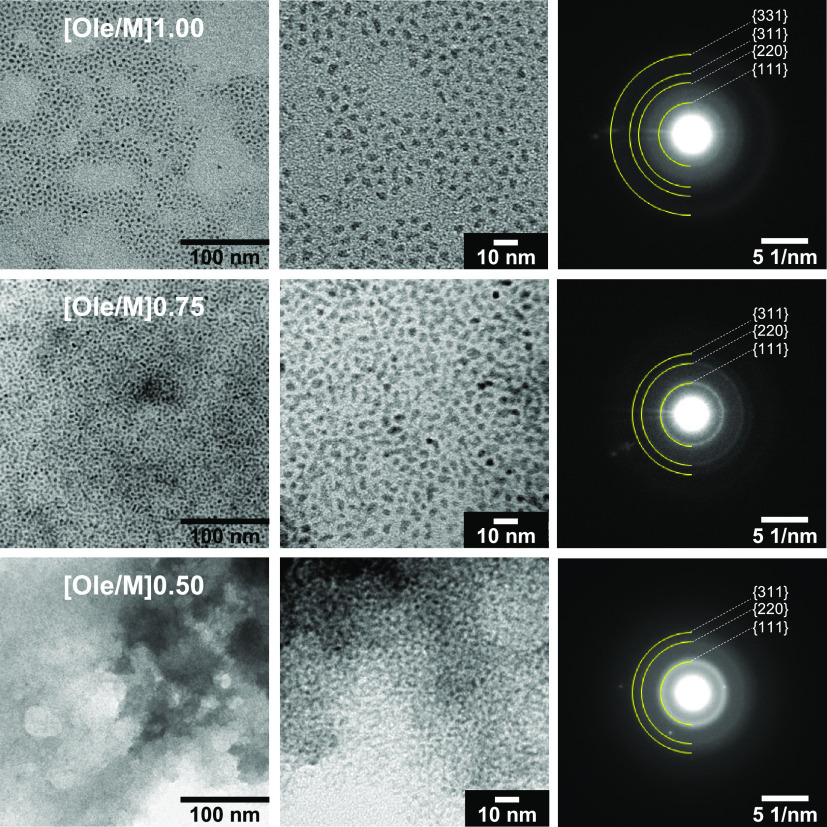
TEM and SAED images of the YSZ NPs synthesized via the hydrothermal method. (Left) Low-magnification, (center) high-magnification, and (right) SAED rings.

TEM was performed to determine the size and dispersed state of the NPs. SAED measurements were used to analyze the crystal structure. Figure 2 shows the TEM and SAED images of the samples. The observed SAED rings in all of the images corresponded to cubic ZrO_2_. Notably, the bright spots in the SAED image of the Ole/M 0.50 sample corresponded to NaCl. The Ole/M 1.00 sample exhibited high dispersibility in cyclohexane owing to the hydrophobic nature of the oleate ligand on the surface. The Ole/M 1.00 sample was composed of triangular particles. In contrast, the Ole/M 0.75 and 0.50 samples possessed agglomerated NPs. The same result was obtained from dynamic light scattering (DLS) measurement (Figure S1 in the Supporting Information).

**Figure 3:**
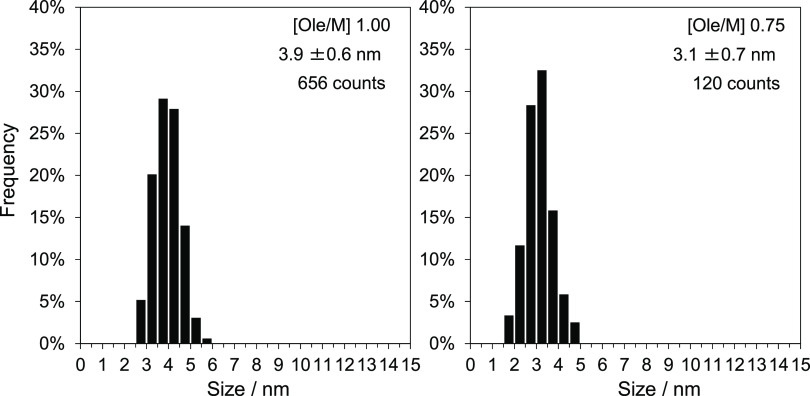
Size distributions of the Ole/M 1.00 and 0.75 samples as determined from the TEM images. The average particle size, standard deviation, and count numbers are shown in the graphs.

The size distributions shown in Figure 3 were determined by counting the particles from the TEM image of the 1.00 and 0.75 samples. The particle size was determined as follows: the area occupied by each particle was approximated to a round shape and its diameter was recorded. The size distribution was then calculated on the basis of the diameter of the round particle. However, for the Ole/M 0.50 sample, the particle size could not be determined because of the presence of strongly agglomerated NPs. The particle size was ∼2 nm by estimation. For the Ole/M 0.75 sample, the grain boundaries were not distinct in the TEM image, thereby affecting the precision of the calculation. More than 100 particles were counted, and the average sizes were 3.9 ± 0.6 and 3.1 ± 0.7 nm for the Ole/M 1.00 and 0.75 samples, respectively. The coefficient of variation (CV), which was calculated from the average particle size and standard deviation to evaluate the variation of size distribution of the samples, was 15% for the Ole/M 1.00 sample, indicating a near monodispersed distribution.

**Figure 4:**
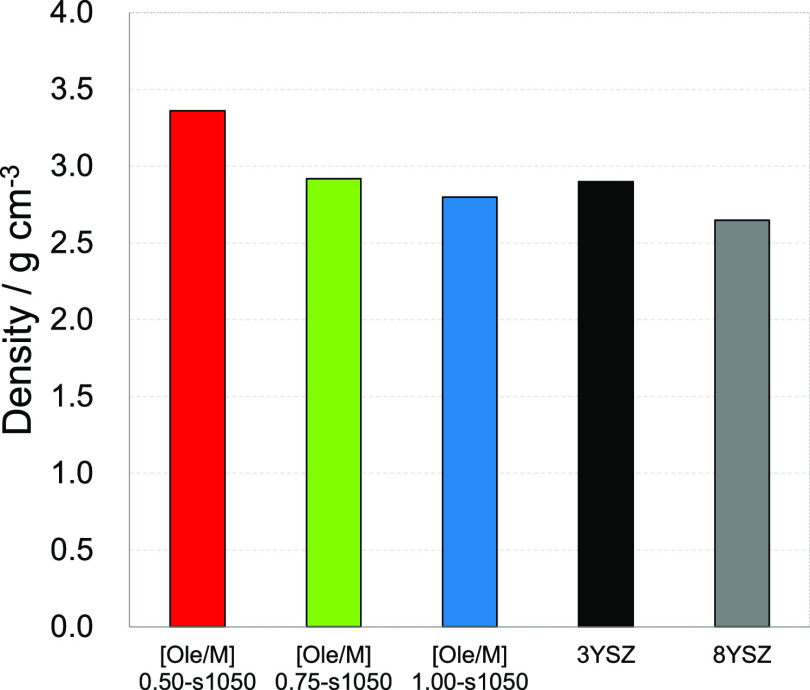
Density of the YSZ NPs with various Ole/M ratios sintered at 1050 °C.

To evaluate the ionic conductivity of the NPs, samples with Ole/M ratios of 0.50, 0.75, and 1.00 were sintered at 1050 °C for 2 h. The density values, as shown in Figure 4, for sintered Ole/M 0.50, 0.75, and 1.00 were 3.36, 2.92, and 2.80 g/cm^3^, respectively, indicating that the Ole/M 0.50 sample exhibited good sinterability. Furthermore, considering the particle size, the high sinterability of the 0.50 Ole/M sample was attributed to its smaller particle size (Figure 2). The densities of commercial 3YSZ and 8YSZ were compared with those of the synthesized YSZ NPs. The density values for 3YSZ and 8YSZ were 2.90 and 2.65 g/cm^3^, respectively; high density was achieved through NP sintering.

**Figure 5:**
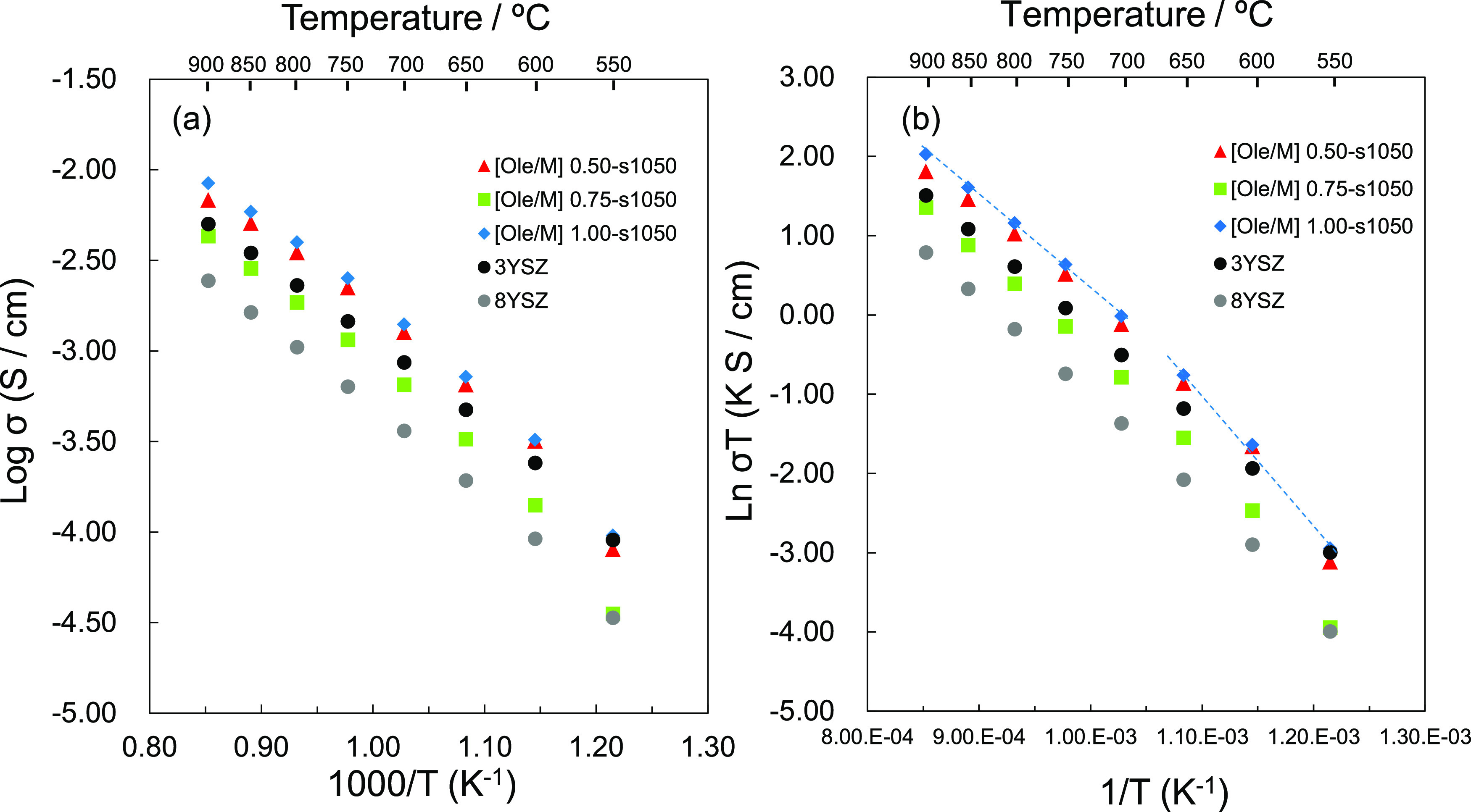
Ionic conductivity of the YSZ NPs with various Ole/M ratios sintered at 1050 °C. (a) Log σ vs 10^3^/T and (b) Ln σ*T* vs 1/*T*.

Figure 5a presents the ionic conductivity of the samples measured via impedance spectroscopy. The disks prepared using NPs with Ole/M ratios of 0.50, 0.75, and 1.00 exhibited ionic conductivities of 2.24, 1.16, and 2.52 mS/cm at 750 °C, respectively. These values were higher than that of commercially available 8YSZ. Unexpectedly, commercial 3YSZ exhibited a higher ionic conductivity than commercial 8YSZ, which can be attributed to the low sinterability of 8YSZ, as shown in Figure 4. The ionic conductivity was highest in the order of the Ole/M 1.00 sample, followed by the Ole/M 0.50 sample, and then the Ole/M 0.75 sample. The result trend was reproducible (a twice duplicated experiment) (see the Supporting Information of Figure S4). Considering duplicated experimental data, the maximum variation of Log σ was 0.38, suggesting that the YSZ nanoparticles synthesized by different Ole/M ratios had different ionic conductivities.

To investigate the factors that enhance the conductivity of YSZ NPs, activation energies were estimated using an Arrhenius plot (Figure 5b). The slope of the Arrhenius plot provided two different activation energy values. The activation energies of the samples in the higher (700–900 °C) and lower (550–650 °C) temperature ranges are summarized in Table 1. No significant difference in the activation energies was observed between the YSZ NPs and commercial YSZ samples in the higher temperature range. However, in the lower temperature range, the YSZ NPs exhibited activation energies higher than those of the commercial YSZ samples (Table 1). The enhancement of ionic conductivity despite the high activation energy is likely because of the increased grain boundary volume or other related factors such as increased ion mobility, changes in the defect concentration in the grain boundary area, or increased contribution of the space charge effect.^14^

**Table 1:** Activation Energy Values (eV) of the YSZ NPs Sintered at 1050 °C in the Higher (700–900 °C) and Lower (550–650 °C) Temperature Ranges

temp	[Ole/M] 0.50-s1050	[Ole/M] 0.75-s1050	[Ole/M] 1.00-s1050	3YSZ	8YSZ
650–550 °C	1.49	1.57	1.43	1.19	1.25
700–900 °C	0.95	1.05	1.00	0.99	1.06

## Discussions

**Figure 6:**
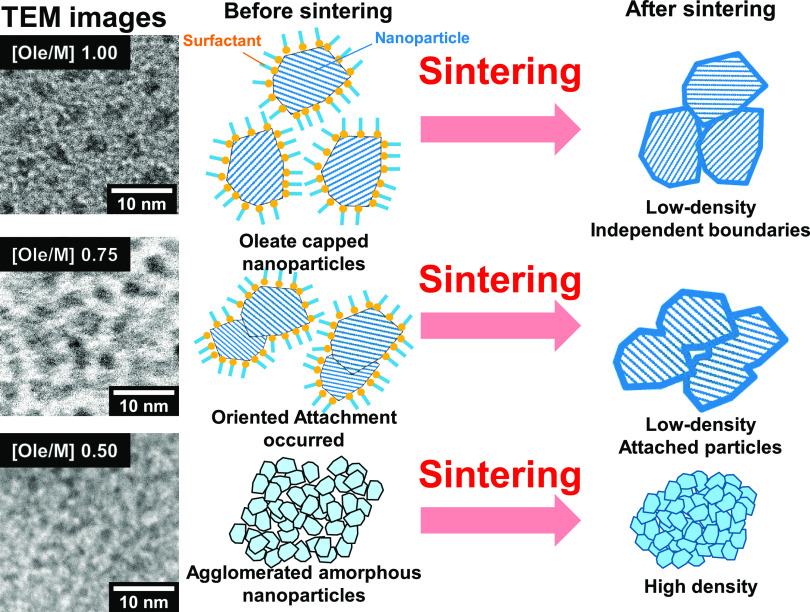
Schematic of the YSZ NPs and boundaries before and after sintering.

**Figure 7:**
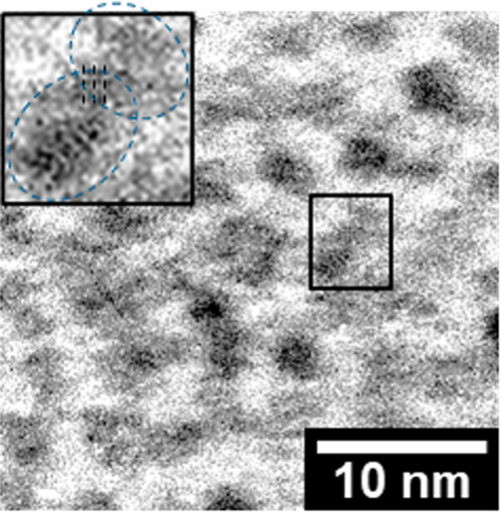
High-resolution TEM image of the Ole/M 0.75 YSZ NPs. The inset image shows a magnified black squared area. The NPs aligned with their crystal facets.

The “independent” surface, which means that a nanoparticle was not connected to other nanoparticles, was considered important in the sintered YSZ NPs. For the sintered YSZ NP samples, the densities were in the order Ole/M 0.50 > 0.75 > 1.00. The Ole/M 0.50 sample exhibited high density because it had the smallest initial particle size. The conductivity order was Ole/M 1.00 > 0.50 > 0.75. The Ole/M 1.00 sample exhibited high conductivity despite its low density, with the key factor being its “independent” boundary. Figure 6 shows schematic illustrations of the YSZ NPs before and after sintering. The Ole/M 1.00 sample was covered with oleate groups, and it was estimated that the boundaries were maintained after sintering. In contrast, the Ole/M 0.75 NPs were attached to each other (as shown in Figure 7) via “oriented attachment,” a process in which crystalline colloidal particles align their crystal facets.^15^

There are many previous studies about the nanoionic effect of YSZ nanoparticles. The most impactful study is by Kosacki et al. They demonstrated that when YSZ thin films were spin-coated on a sapphire substrate and then sintered at 1000 °C, the grain size of the sample was around 20 nm and the conductivity at 900 °C was Log σ: −1.2.^5^ This value is comparable to that of densely sintered YSZ and is 0.5 higher than our sample. The enhanced conductivity of nanocrystals is speculated to be due to a reduction in the activation energy. The activation energy was reduced from 1.23 to 0.93 eV. This phenomenon is probably attributed to the expanded interface region of nanocrystalline materials and the unique defect thermodynamics determining the hopping energy of oxygen ions. However, in another study by Durá, it was shown that when physically milled YSZ nanoparticles were used to elucidate the size dependency and conductivity relationship, the ionic conductivity of a pellet compressed from 17 nm particles tended to be smaller than other sizes.^7^ This suggests that simple particle size does not solely influence ionic conductivity, but it is also significantly affected by the synthesis method. Generally, samples sintered at 1300–1500 °C tend to show higher ionic conductivities.^16,17^ Their data were observed around Ln (σ*T*) of 4 at 900 °C, which translates to Log σ of approximately −1.2. Additionally, in a review by Mohd Affandi et al., one advantage mentioned for nanosized materials is that after sintering nanosized particles, more grain boundaries might remain, which can positively influence the oxide ionic conductivity.^18^ From the above discussion, we infer that the difference observed in our YSZ nanoparticles is potentially due to the remaining grain boundaries. Sintering dispersed particles rather than merely agglomerated ones might increase the possibility of having more grain boundaries.

## Conclusions

In this study, YSZ NPs with an oleate group and various Ole/M ratios were synthesized by the hydrothermal method. The synthesized YSZ NPs were then sintered and characterized. Both the NPs and sintered YSZ exhibited the cubic ZrO_2_ crystal phase. The YSZ NP sizes were 3.9 ± 0.6 and 3.1 ± 0.7 nm at Ole/M ratios of 1.00 and 0.75, respectively. The 1.00 Ole/M sample exhibited high dispersibility in cyclohexane and a CV of 15%, which indicates a nearly monodispersed distribution. The densities of the Ole/M 0.50, 0.75, and 1.00 sintered samples were 3.36, 2.92, and 2.80 g/cm^3^, respectively. The 0.50 Ole/M sample demonstrated good sinterability, which is attributed to its small particle size. The disks prepared with the Ole/M 0.50, 0.75, and 1.00 sintered samples exhibited ionic conductivities of 2.24, 1.16, and 2.52 mS/cm at 750 °C, respectively; these values were higher than that of commercially available 8YSZ. The activation energies were calculated by the Arrhenius plot, and the YSZ NPs exhibited higher activation energies than commercial YSZ in the lower temperature range.

## Supporting Information Available

**Figure S1**. Size distributions of the YSZ NPs synthesized via the hydrothermal method, as obtained from DLS measurements. **Figure S2**. Cross sectional SEM images of sintered YSZ (Ole/M 0.50, 0.75, 1.00) and commercial 8YSZ. **Figure S3**. Electrochemical impedance spectroscopy (EIS) plot of the sintered YSZ nanoparticles and reference YSZ. **Figure S4**. Ionic conductivity of different batches of YSZ NPs with various Ole/M ratios sintered at 1050 °C. (a) Log σ vs 10^3^/*T* and (b) Ln σ*T* vs 1/*T* (PDF)

## References

[ref1] Zakaria Z.; Abu Hassan S. H.; Shaari N.; Yahaya A. Z.; Boon Kar Y. A Review on Recent Status and Challenges of Yttria Stabilized Zirconia Modification to Lowering the Temperature of Solid Oxide Fuel Cells Operation. Int. J. Energy Res. 2020, 44 (2), 631–650. 10.1002/er.4944.

[ref2] Ploner A.; Hagen A.; Hauch A. Classical Statistical Methodology for Accelerated Testing of Solid Oxide Fuel Cells. J. Power Sources 2018, 395, 379–385. 10.1016/j.jpowsour.2018.05.034.

[ref3] Zakaria Z.; Kamarudin S. K. Advanced Modification of Scandia-Stabilized Zirconia Electrolytes for Solid Oxide Fuel Cells Application—A Review. Int. J. Energy Res. 2021, 45 (4), 4871–4887. 10.1002/er.6206.

[ref4] Shi H.; Su C.; Ran R.; Cao J.; Shao Z. Electrolyte Materials for Intermediate-Temperature Solid Oxide Fuel Cells. Prog. Nat. Sci. Mater. Int. 2020, 30 (6), 764–774. 10.1016/j.pnsc.2020.09.003.

[ref5] Kosacki I. Electrical Conductivity of Nanocrystalline Ceria and Zirconia Thin Films. Solid State Ionics 2000, 136–137 (1–2), 1225–1233. 10.1016/S0167-2738(00)00591-9.

[ref6] Bagchi B.; Basu R. N. A Simple Sol–Gel Approach to Synthesize Nanocrystalline 8 Mol% Yttria Stabilized Zirconia from Metal-Chelate Precursors: Microstructural Evolution and Conductivity Studies. J. Alloys Compd. 2015, 647, 620–626. 10.1016/j.jallcom.2015.06.082.

[ref7] Durá O. J.; López de la Torre M. A.; Vázquez L.; Chaboy J.; Boada R.; Rivera-Calzada A.; Santamaria J.; Leon C. Ionic Conductivity of Nanocrystalline Yttria-Stabilized Zirconia: Grain Boundary and Size Effects. Phys. Rev. B 2010, 81 (18), 184301 10.1103/PhysRevB.81.184301.

[ref8] Evans A.; Bieberle-Hütter A.; Rupp J. L. M.; Gauckler L. J. Review on Microfabricated Micro-Solid Oxide Fuel Cell Membranes. J. Power Sources 2009, 194 (1), 119–129. 10.1016/j.jpowsour.2009.03.048.

[ref9] Makinose Y.; Taniguchi T.; Katsumata K.; Okada K.; Matsushita N. Facet Control of Ceria Nanocrystals Synthesized by an Oleate-Modified Hydrothermal Method. Adv. Powder Technol. 2016, 27 (1), 64–71. 10.1016/j.apt.2015.10.004.

[ref10] Taniguchi T.; Watanabe T.; Katsumata K.; Okada K.; Matsushita N. Synthesis of Amphipathic YVO_4_ :Eu ^3+^ Nanophosphors by Oleate-Modified Nucleation/Hydrothermal-Growth Process. J. Phys. Chem. C 2010, 114 (9), 3763–3769. 10.1021/jp908959t.

[ref11] Ohno Y.; Tomita K.; Komatsubara Y.; Taniguchi T.; Katsumata K.; Matsushita N.; Kogure T.; Okada K. Pseudo-Cube Shaped Brookite (TiO_2_) Nanocrystals Synthesized by an Oleate-Modified Hydrothermal Growth Method. Cryst. Growth Des. 2011, 11 (11), 4831–4836. 10.1021/cg2006265.

[ref12] Schneider C. A.; Rasband W. S.; Eliceiri K. W. NIH Image to ImageJ: 25 Years of Image Analysis. Nat. Methods 2012, 9 (7), 671–675. 10.1038/nmeth.2089. 22930834 PMC5554542

[ref13] Seto Y.; Ohtsuka M. ReciPro: Free and Open-Source Multipurpose Crystallographic Software Integrating a Crystal Model Database and Viewer, Diffraction and Microscopy Simulators, and Diffraction Data Analysis Tools. J. Appl. Crystallogr. 2022, 55 (2), 397–410. 10.1107/S1600576722000139.

[ref14] Vijaya Lakshmi V.; Bauri R.; Gandhi A. S.; Paul S. Synthesis and Characterization of Nanocrystalline ScSZ Electrolyte for SOFCs. Int. J. Hydrogen Energy 2011, 36 (22), 14936–14942. 10.1016/j.ijhydene.2011.02.139.

[ref15] Salzmann B. B. V.; Van Der Sluijs M. M.; Soligno G.; Vanmaekelbergh D. Oriented Attachment: From Natural Crystal Growth to a Materials Engineering Tool. Acc. Chem. Res. 2021, 54 (4), 787–797. 10.1021/acs.accounts.0c00739. 33502844 PMC7893701

[ref16] Mæland D.; Suciu C.; Wærnhus I.; Hoffmann A. C. Sintering of 4YSZ (ZrO_2_ + 4 mol % Y_2_O_3_) Nanoceramics for Solid Oxide Fuel Cells (SOFCs), Their Structure and Ionic Conductivity. J. Eur. Ceram. Soc. 2009, 29 (12), 2537–2547. 10.1016/j.jeurceramsoc.2009.03.013.

[ref17] Chervin C. N.; Clapsaddle B. J.; Chiu H. W.; Gash A. E.; Satcher Joe H.; Kauzlarich S. M. Aerogel Synthesis of Yttria-Stabilized Zirconia by a Non-Alkoxide Sol–Gel Route. Chem. Mater. 2005, 17 (13), 3345–3351. 10.1021/cm0503679.

[ref18] Mohd Affandi N. S.; Osman N. Short Review on Global Trends in SOFC Scenario and Future Perspective. Mater. Today Proc. 2022, 66, 3981–3984. 10.1016/j.matpr.2022.04.824.

