# Supplementary material for: Ionic Conductivity of Electrolytes Composed of Oleate-Capped Yttria-Stabilized Zirconia Nanoparticles
Source: ACS Omega. 2023 Dec 7;8(51):48728–34. doi: 10.1021/acsomega.3c05368 (PMC10753559; doi:10.1021/acsomega.3c05368)
Supplement: Supplementary file 1 — ao3c05368_si_001.pdf [file ao3c05368_si_001.pdf]

Ionic Conductivity of Electrolytes Composed of Oleate-Capped Yttria-Stabilized Zirconia Nanoparticles

Yuki Makinose[1], Tetsuya Yamada[2*], Yuta Kubota[3]

[1] Graduate School of Natural Science and Technology, Shimane University, 1060 Nishikawatsu-cho, Matsue 690–8504, Japan

[2] Laboratory for Future Interdisciplinary Research of Science and Technology, Tokyo Institute of Technology, 4259 Nagatsuta, Midori, Yokohama, Kanagawa 226-8503, Japan

[3] Department of Materials Science and Engineering, School of Materials and Chemical Technology, Tokyo Institute of Technology, 2-12-1 Ookayama, Meguro, Tokyo 152-8550, Japan

Supporting Information

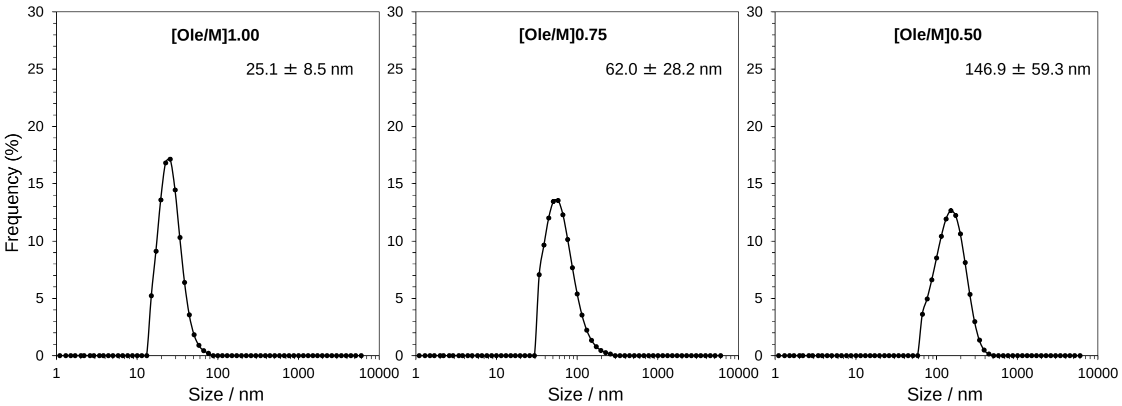

Figure S1. Size distributions of the YSZ NPs synthesized via the hydrothermal method, as obtained from DLS measurements. The average particle size and standard deviation are shown on the graphs.

Dynamic light scattering (DLS) measurements were conducted for the size distribution of the nanoparticles in solution using a Horiba LB-550 system. The dispersion concentration was higher than the one of TEM dispersion. Thus, the particle sizes of DLS measurement were higher than those of TEM images. The average particle size, shown in Figure S1, increased with decreasing the Ole/M ratio. The result indicates that the nanoparticles agglomerated with low Ole/M, especially strongly agglomerated with Ole/M of 0.50.

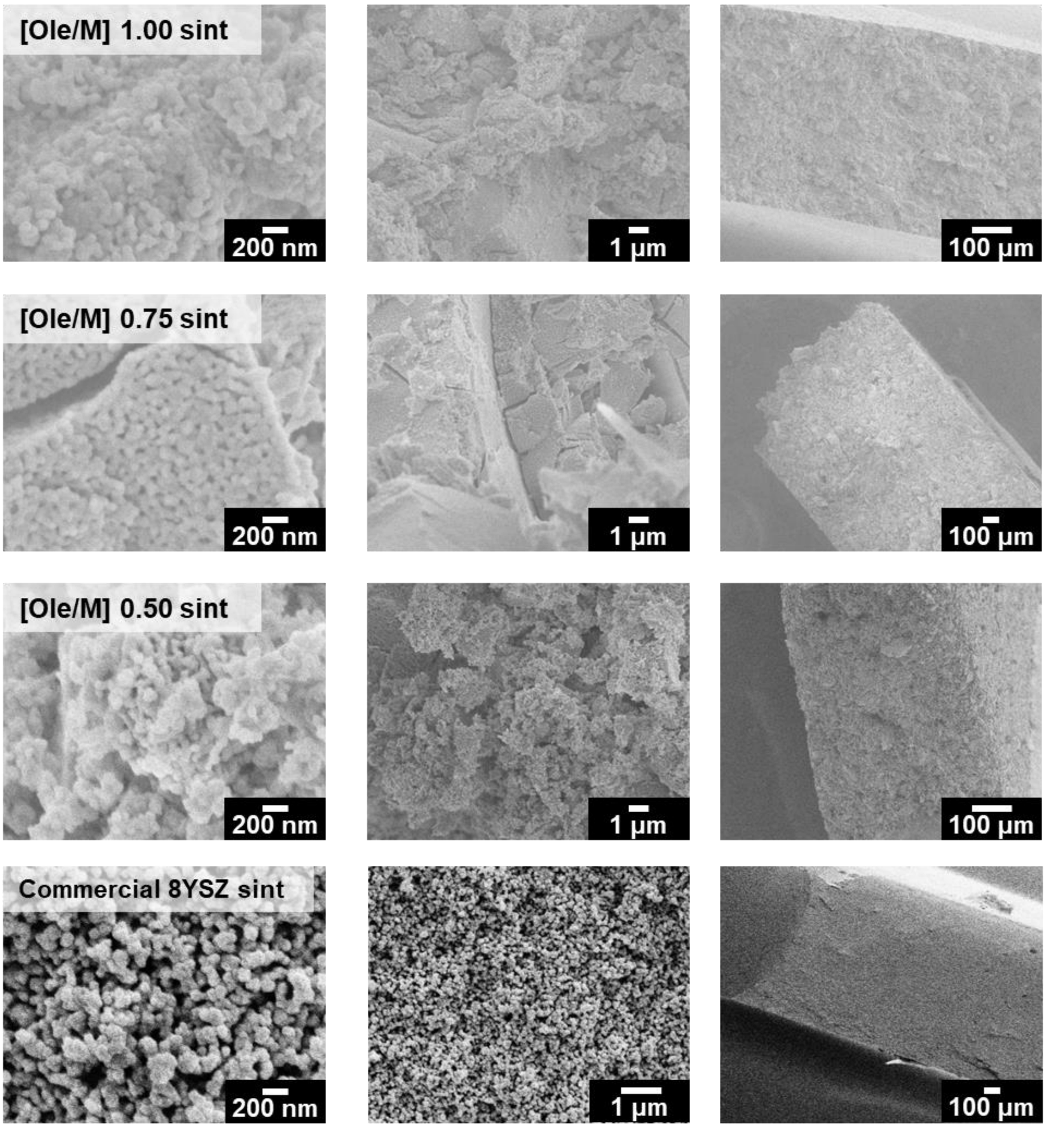

Figure S2. Cross sectional SEM images of sintered YSZ (Ole/M 0.50, 0.75, 1.00) and commercial 8YSZ.

Cross-sectional Scanning Electron Microscope (SEM) images are shown in Fig. S2. The images of sintered YSZ NPs were observed using a Field Emission Scanning Electron Microscope (FE-SEM), JSM-7001FA (JEOL, Japan). The sample was divided into small pieces for cross-sectional observation. It was then fixed onto the SEM stage using Ag paste for fixation. The SEM stage was gold-sputtered to a thickness of approximately 20Å. Please note that the SEM images of sintered Ole/M 0.75 samples and commercial 8YSZ were prepared using 500 mg.

The pellets are formed by agglomerated nanoparticles and has voids within them. The particle sizes are about 50-120 nm. Single nanoparticles seemed to consist of small-sized particles aggregated together. Nanoparticle size can vary depending on which portion is considered as a particle. The presence of numerous small and large pores was observed. We could not calculate the porosity due to the complex nature of the pores in the sintered YSZ nanoparticles.

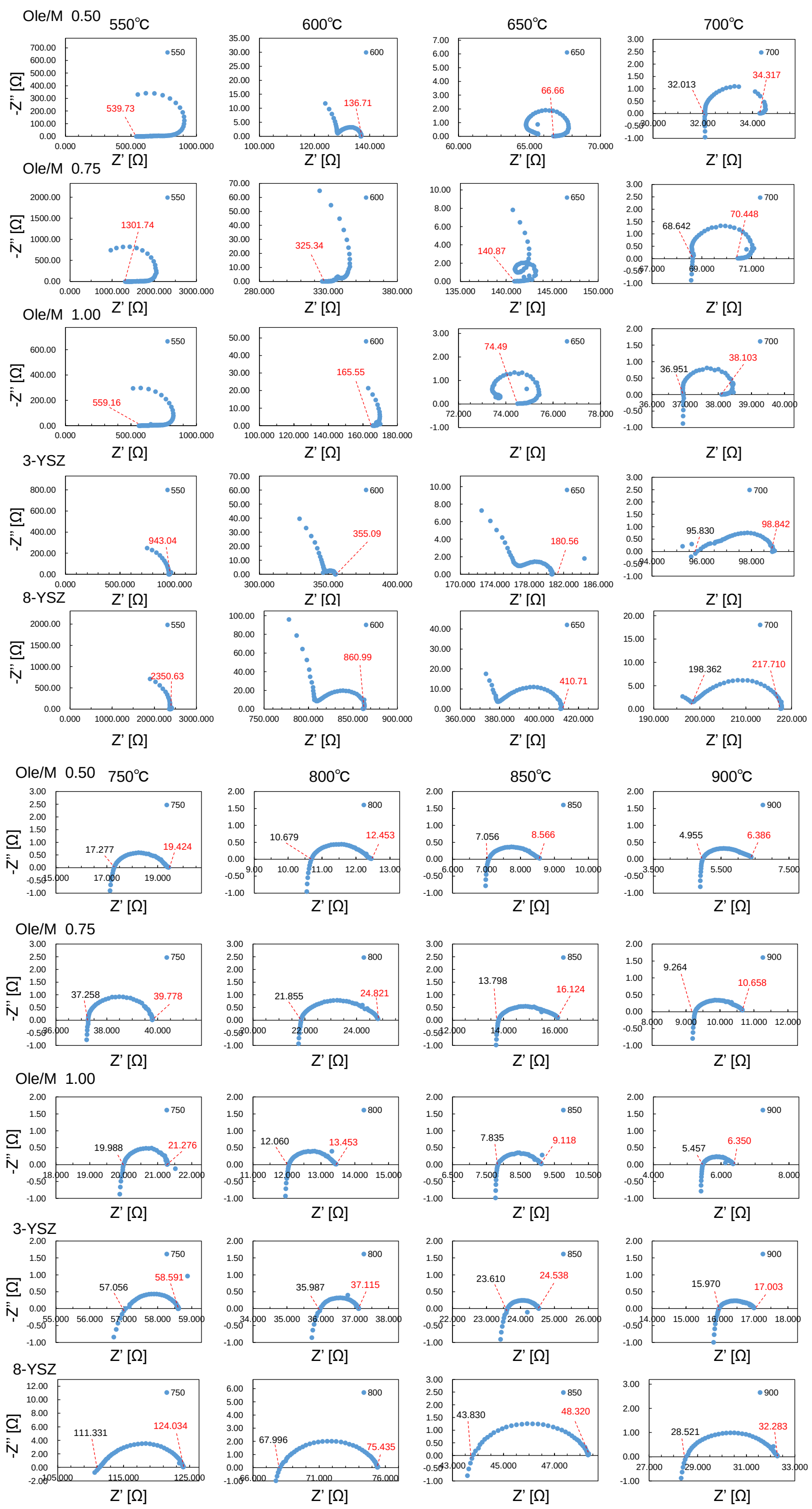

Figure S3. Electrochemical impedance spectroscopy (EIS) plot of the sintered YSZ nanoparticles and refence YSZ.

Electrochemical impedance spectroscopy (EIS) plots are shown in Fig. S3. The curve is composed of semi-circles relating to bulk and grain resistance. This study focused on analyzing the total resistance. The resistance at each temperature was selected from the high resistance side of the intersection point between the EIS plot and real resistance axis, or the value closest to the intersection point. The thickness and diameter of each YSZ sample are shown in Table S1. Please note that the commercial product has approximately twice the thickness of the synthesized nanoparticle sample.

Table S1. Thickness and diameter of sintered YSZ

Sample name	Ole/M /M 0.50-1050	Ole/M /M 0.75-1050	Ole/M /M 1.00-1050	3YSZ-1050	8YSZ-1050
t(mm)	0.532	0.575	0.624	1.226	1.217
ϕ (mm)	12.475	12.620	12.181	13.503	13.995

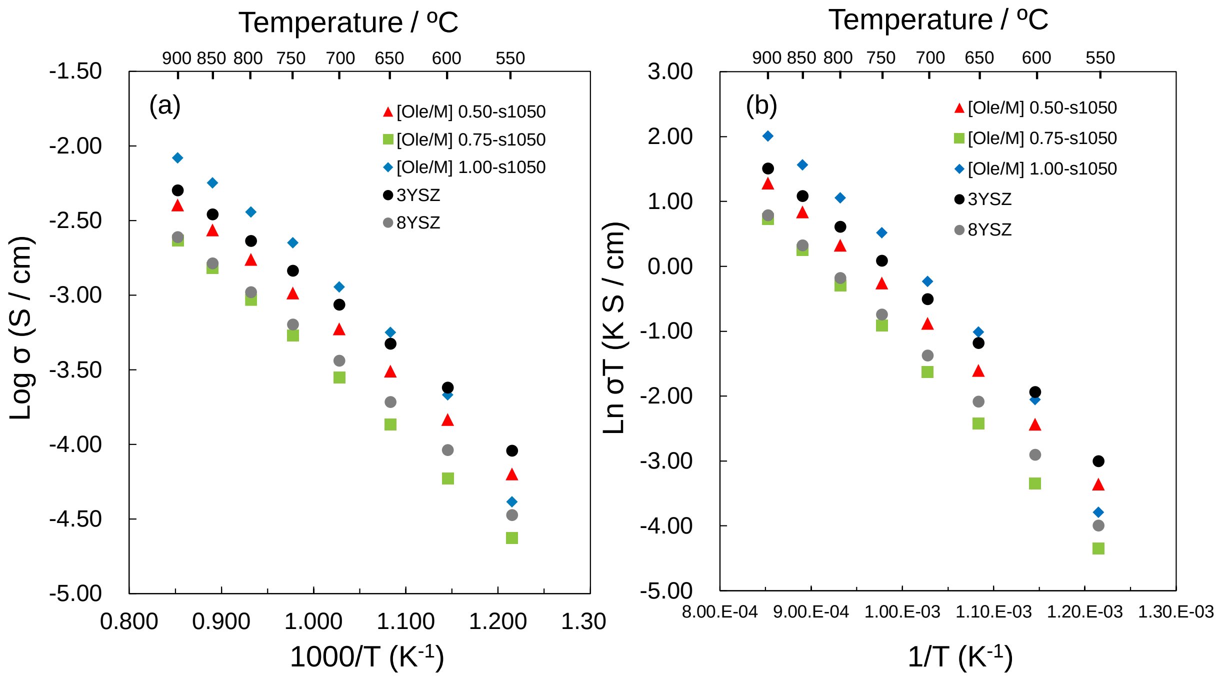

Figure S4. Ionic conductivity of different batch YSZ NPs with various Ole/M ratios sintered at 1050 °C. (a) Log σ vs 10^3/T. and (b) Ln σT vs 1/T.

Fig. S4 shows the result of the replication experiment. The ionic conductivity was highest in the order of the Ole/M 1.00, followed by the Ole/M 0.50, and then 0.75. Upon measuring twice, this order was consistently maintained.
